# Coronary Vasospasm While Treating Supraventricular Tachycardia: Is Adenosine Really to Blame?

**DOI:** 10.1155/2013/897813

**Published:** 2013-04-10

**Authors:** Henry C. Quevedo, Jerson Munoz-Mendoza, Veronica Pinto Miranda, Rafael F. Sequeira

**Affiliations:** ^1^Tulane University, Heart and Vascular Institute, New Orleans, LA 70118, USA; ^2^University of Miami, Miller School of Medicine, Division of Internal Medicine, Miami, FL 33136, USA; ^3^Department of Medicine, University of Miami/Jackson Memorial Hospital, 1611 NW 12th Avenue, Central Building, Room 600D, Miami, FL 33136, USA; ^4^University of Miami, Miller School of Medicine, Division of Cardiovascular Medicine, Miami, FL 33136, USA

## Abstract

Coronary artery spasm has been reported during adenosine stress testing. Herein, we describe a transient ST-segment elevation following adenosine therapy for supraventricular tachycardia. A 38-year-old male presented to the emergency department with palpitations. Electrocardiogram showed supraventricular tachycardia with short RP interval. Vagal maneuvers were unsuccessful. Adenosine was then administered in two successive injections of 6 and 12 mg dosages, respectively. A subsequent 12-lead electrocardiogram revealed ST-segment elevation in inferior leads with reciprocal changes. Coronary angiography disclosed nonobstructive coronary disease. A postprocedure electrocardiogram exhibited normal sinus rhythm with nonspecific T wave abnormalities. Cardiac biomarkers were elevated with a peak troponin I of 0.32. Echocardiogram depicted bicuspid aortic valve and normal systolic function. Electrophysiological study revealed a concealed left accessory pathway and successful radiofrequency ablation was performed. Given the dynamic changes in the electrocardiogram, we hypothesize that this event was most likely a coronary vasospasm. The mechanism of coronary spasm following adenosine injection remains uncertain. Potential mediators include K_ATP_ channels and adenosine-2 receptors.

## 1. Introduction

Adenosine is a frequently used pharmacologic stress agent in myocardial perfusion imaging and supraventricular tachyarrhythmia (SVT) termination, also known as atrioventricular blocking effect. Its safety profile is well established, and most of its side effects are mild and transient [1]. Coronary vasospasm has been reported during or after adenosine stress test, which may lead to seriously adverse outcomes [1]. Herein, we present a case of ST-segment elevation myocardial infarction following an intravenous bolus dose of adenosine for SVT termination.

## 2. Case Presentation

A 38-year-old Hispanic male, without known cardiovascular diseases presented to the emergency room complaining of two-day history of intermittent palpitations. He also stated having a three-hour pressure-like epigastric discomfort with radiation to the right upper quadrant. The pain started while lifting heavy objects at work and continued intermittently. There was no history of syncopal or presyncopal episodes. His past medical history disclosed multiple episodes of palpitations since the age of 20, but no associated chest pain, a syncopal episode related to exercise a year earlier and negative history of illicit drugs, tobacco, or alcohol intake. Importantly, his father died at the age of 57 due to a massive myocardial infarction.

**Figure 1:**
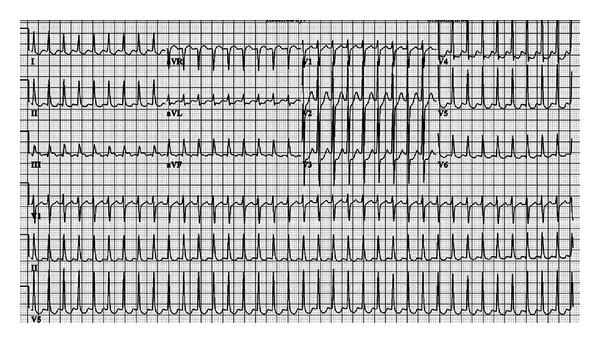
12-lead ECG on admission exhibiting supraventricular tachycardia with short RP interval.

**Figure 2:**
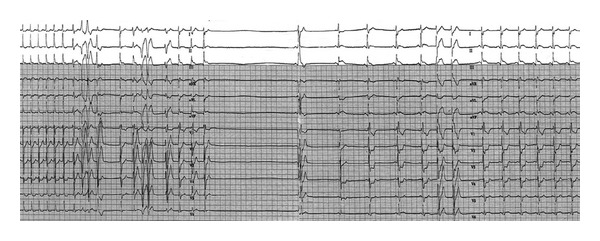
Continuous ECG recording depicts the response to the second dose of adenosine (12 mg). After the initial delayed conduction in the atrioventricular node and sinus node, sinus pause arose. Sinus rhythm then resumed and ST-segment elevation in leads II, III, and aVF with ST-segment depression in leads I, aVL, and V1-V3 are seen.

**Figure 3:**
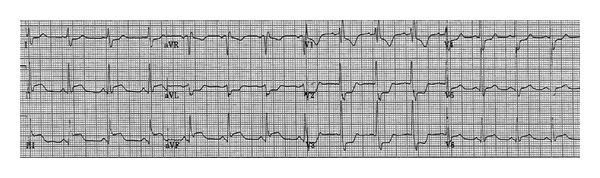
12-lead ECG is depicting an ST-segment elevation in inferior leads and reciprocal changes in lead I, and morphology of right bundle branch block (rsR') in lead V1 with ST-segment depressions in V1-V3 is observed after the adenosine injection.

**Figure 4:**
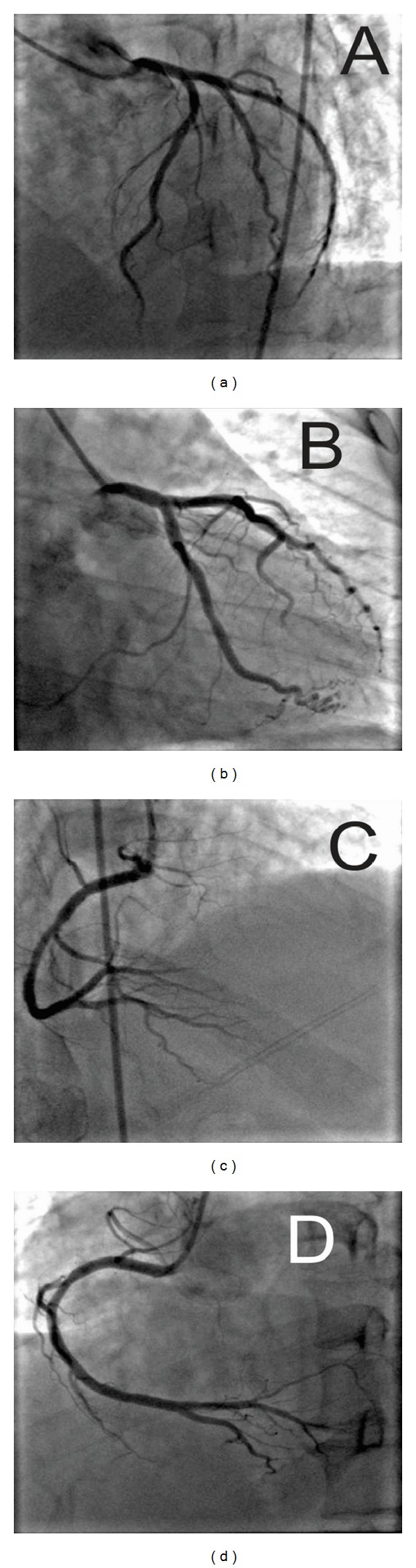
Conventional angiographic projections are shown to evaluate the left anterior descending artery, circumflex artery (cranial and oblique projections, (a) and (b), resp.), and right coronary artery (cranial and oblique, (c) and (d), resp.). No significant coronary disease was seen.

Upon arrival, blood pressure was 100 mm Hg systolic and 60 mm Hg diastolic; he was tachycardiac with a heart rate of 220 beats per minute. Apart from tachycardia, cardiovascular examination and the rest of physical exam were unremarkable. An electrocardiogram (ECG) showed narrow complex tachycardia with a ventricular rate of 220 beats per minute (Figure 1). Presumed ST-segment elevation <1 mm was noted only in lead III during tachycardia. Vagal maneuvers were attempted to convert the rhythm but were unsuccessful. Subsequently, successive intravenous boluses injection of adenosine at doses of 6 and 12 mg were administered with successful conversion to sinus rhythm (Figure 2). As the rhythm returned to sinus, significant ST segment elevation in the inferior leads and reciprocal changes as well as right bundle branch block were appreciated (Figure 3). At this point, the patient was still having epigastric discomfort, and repeated troponin I was elevated with a maximum value of 0.32. Therapy for acute coronary syndrome was started including aspirin, clopidogrel, statins, metoprolol, and intravenous nitroglycerine. Anticoagulation was not administered since the patient was emergently transported to the catheterization laboratory within 30 minutes. When the patient arrived to the catheterization laboratory, the ST-segment elevation had resolved with marked relief of his symptoms. Coronary angiography documented non-obstructive coronary disease. Also left ventricular angiography depicted normal systolic function and no wall motion abnormalities (Figures 4(a)–4(d)). The interventional team felt that a provocation test for coronary artery spasm was not safe in these circumstances. The patient then continued with nitroglycerine drip and his epigastric discomfort resolved when the heart rate decreased to less than 100/minute in the Coronary Care Unit. Transthoracic echocardiography performed the morning after revealed preserved left ventricular ejection fraction with no segmental wall motion abnormalities and a bicuspid aortic valve but no aortic stenosis.

**Figure 5:**
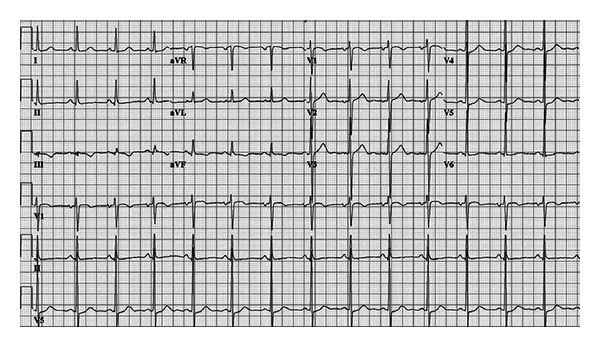
Follow-up 12-lead ECG exhibited normal sinus rhythm.

Once our patient was clinically stable, an electrophysiological study was performed revealing a concealed left sided accessory pathway and he underwent successful radiofrequency ablation of the slow pathway of the atrioventricular nodal reentrant tachycardia. ECG after procedure showed normal sinus rhythm with nonspecific ST-T wave changes (Figure 5). Outpatient followup was missed, but the patient returned nine months later to the emergency department for an abscess on his hand that required local debridement and intravenous antibiotics. No further episodes of chest pain, ECG changes, or arrhythmias were documented during his second hospitalization.

## 3. Discussion

Adenosine usually has a vasodilator effect in the coronary microcirculation; however this case demonstrated an unusual complication of intravenous administration of adenosine, coronary vasospasm. 

After administration of adenosine, the presence of persistent epigastric pain along with ST-segment elevation with reciprocal changes that were reverted by nitrates was very suggestive of an ischemic event, in which cardiac catheterization did not show significant obstructive coronary artery disease as the cause of the patient symptoms. Importantly, the patient was on sinus rhythm by the time his ECG changes developed. Coronary artery vasospasm was the most likely explanation of the transient ECG changes. His ECG changes, including the presence of right bundle branch block, possibly reflect the abnormality of more than one coronary territory pointing towards a transient diffuse vasospasm. 

Nevertheless, our patient did not present as a typical case of Prinzmetal variant angina, because the initial symptom of our patient was palpitations and no chest pain, indicating that coronary vasospasm was not the initial event as in cases of Prinzmetal variant angina. In addition, neither the classic associated risk factors for Prinzmetal angina nor any triggering events for the vasospastic phenomenon such as alcohol, nicotine, cocaine, or iced drinks ingestion were present [2]. Given the fact that our patient did not show wall motion abnormalities in the left ventricular angiography, stress induced cardiomyopathy (tako-tsubo) would be also less likely to be proposed as a diagnosis.

A number of cases of coronary vasospasm during [3, 4] or after [4–6] an adenosine stress test have been reported; however adenosine induced coronary artery vasospasm provoked by intravenous administration for SVT termination has only recently been reported [7], and our patient would constitute the second reported case worldwide. Coronary artery spasm results from the interaction of 2 components: (1) usually localized, but sometimes diffuse, abnormality of a coronary artery that makes it hyperreactive to vasoconstrictor stimuli and (2) a vasoconstrictor stimulus able to induce the spasm at the level of the hyperreactive coronary segment [8]. Accordingly, coronary artery spasm would potentially affect predisposed individuals with at least one hyperreactive point/area in a coronary artery. 

The biological actions of adenosine are exerted via activation of four receptors (A1, A2a, A2b, and A3) which, then, act through a second messenger system. Scenarios for the pharmacological actions of the adenosine receptors include atrioventricular node blockade (A1), vasodilation (A2a), ischemic preconditioning (A2b), and mast cell degranulation (A3) [9]. Adenosine also directly stimulates the ATP-sensitive potassium (K_ATP_) channels in the coronary arterial smooth muscle cell [10] which regulate vascular tone [11]. The exact mechanism that explains the vasoconstrictor properties of adenosine is unknown; however it is believed to be related to structural changes in the K_ATP_ channel in the coronary arterial smooth muscle cell, as demonstrated in experimental models [11].

In conclusion, adenosine typically has a vasodilator effect in the coronary microcirculation; however this case demonstrated an unusual complication of intravenous administration of adenosine, coronary vasospasm. Adenosine usually features a safe pharmacological profile; however possible mutations in the downstream signaling pathway could potentially lead to coronary artery vasospasm in a small amount of predisposed individuals.
